# Predicting behavioural response to TDCS in chronic motor stroke^☆^

**DOI:** 10.1016/j.neuroimage.2013.05.096

**Published:** 2014-01-15

**Authors:** Jacinta O'Shea, Marie-Hélène Boudrias, Charlotte Jane Stagg, Velicia Bachtiar, Udo Kischka, Jakob Udby Blicher, Heidi Johansen-Berg

**Affiliations:** a Oxford Centre for Functional MRI of the Brain (FMRIB), Nuffield Department of Clinical Neurosciences, University of Oxford, John Radcliffe Hospital, Headley Way, Headington, Oxford OX3 9DU, UK; b Oxford Centre for Enablement, Nuffield Orthopaedic Centre, Oxford OX3 7HE, UK

**Keywords:** Motor stroke, Plasticity, TDCS, Brain stimulation, Magnetic resonance spectroscopy, GABA

## Abstract

Transcranial direct current stimulation (TDCS) of primary motor cortex (M1) can transiently improve paretic hand function in chronic stroke. However, responses are variable so there is incentive to try to improve efficacy and or to predict response in individual patients. Both excitatory (Anodal) stimulation of ipsilesional M1 and inhibitory (Cathodal) stimulation of contralesional M1 can speed simple reaction time. Here we tested whether combining these two effects simultaneously, by using a bilateral M1–M1 electrode montage, would improve efficacy. We tested the physiological efficacy of Bilateral, Anodal or Cathodal TDCS in changing motor evoked potentials (MEPs) in the healthy brain and their behavioural efficacy in changing reaction times with the paretic hand in chronic stroke. In addition, we aimed to identify clinical or neurochemical predictors of patients' behavioural response to TDCS. There were three main findings: 1) unlike Anodal and Cathodal TDCS, Bilateral M1–M1 TDCS (1 mA, 20 min) had no significant effect on MEPs in the healthy brain or on reaction time with the paretic hand in chronic stroke patients; 2) GABA levels in ipsilesional M1 predicted patients' behavioural gains from Anodal TDCS; and 3) although patients were in the chronic phase, time since stroke (and its combination with Fugl–Meyer score) was a positive predictor of behavioural gain from Cathodal TDCS. These findings indicate the superiority of Anodal or Cathodal over Bilateral TDCS in changing motor cortico-spinal excitability in the healthy brain and in speeding reaction time in chronic stroke. The identified clinical and neurochemical markers of behavioural response should help to inform the optimization of TDCS delivery and to predict patient outcome variability in future TDCS intervention studies in chronic motor stroke.

## Highlights

• Ipsilesional M1 GABA levels predict motor gains from Anodal TDCS in chronic stroke.
• Time since stroke and Fugl–Meyer score jointly predict response to Cathodal TDCS.
• Bilateral motor cortex TDCS did not reliably change motor evoked potentials.
• Bilateral motor cortex TDCS did not reliably change manual reaction time.

## Introduction

Stroke is a leading cause of life-long disability (Adamson et al., 2004). Treatments that can improve function and reduce disability once patients have reached the chronic phase are limited. Transcranial direct current stimulation (TDCS) is a non-invasive brain stimulation technique with demonstrated potential for alleviating motor deficits in chronic stroke. Anodal stimulation increases while Cathodal stimulation decreases the excitability of primary motor cortex (M1) (Nitsche and Paulus, 2000). Both Anodal stimulation of ipsilesional M1 and Cathodal stimulation of contralesional M1 have been shown to induce short-lived improvements in paretic hand function after stroke (Fregni et al., 2005; Hummel et al., 2006). Both interventions increase task-related functional motor activity in ipsilesional M1 and in a number of inter-connected secondary motor areas (Stagg et al., 2012). Functional gains are thought to arise by two different routes: Anodal TDCS directly boosts excitability of the ipsilesional M1/corticospinal tract (Hummel et al., 2006), whereas Cathodal TDCS is thought to reduce inter-hemispheric inhibition from contralesional-to-ipsilesional M1 (Duque et al., 2005; Murase et al., 2004). On the question of which of these two stimulation strategies should have greater therapeutic efficacy for a given individual, the answer is unclear, partly because Anodal and Cathodal TDCS are rarely compared directly in the same patients (although see Fregni et al., 2005; Stagg et al., 2012).

In principle, Anodal TDCS, by increasing excitability in ipsilesional M1/corticospinal tract, offers the potential to improve hand weakness in any hemiparetic patient. In practice, however, its actual therapeutic utility for a given patient is likely to be limited by the residual integrity of the stimulated cortex and associated motor corticospinal tract (Zhu et al., 2010). A high degree of damage, reflected in an absence or reduction in amplitude of motor-evoked potentials (MEPs) (Heald et al., 1993; Ward et al., 2006), or in significant asymmetry in diffusion tensor imaging (DTI) measures (Lotze et al., 2012; Stinear, 2010; Stinear et al., 2007), predicts poor potential for functional recovery, limiting the likely efficacy of rehabilitative interventions aimed at restoring function within that pathway.

Evidence predicts that Cathodal TDCS, which aims to suppress contralesional motor activity, may prove beneficial for some patients, but detrimental for others, depending on the functional significance of that contralesional activity in driving paretic hand use for a given individual. When stroke patients move their paretic hand, there is abnormally increased activity in motor regions of the contralesional hemisphere (Ward et al., 2003a). The more damage to the ipsilesional corticospinal tract, the greater the extent of abnormal bilateral activity. Bilateral activity is prominent in poorly-recovered patients, and less prominent in those who make a good recovery, in whom brain activity tends to re-normalize to the ipsilesional hemisphere over time (Ward et al., 2003b). The functional status of contralesional motor activity is critical for predicting the likely impact of suppressive Cathodal TDCS: in less impaired patients contralesional activity tends to contribute to disability, whereas in more impaired patients it can help to drive compensatory use of the paretic hand (Fridman et al., 2004; Johansen-Berg et al., 2002; Lotze et al., 2006). Hence, the effect of Cathodal TDCS, which aims to suppress contralesional motor activity, is likely to vary across individuals, facilitating or impeding paretic hand use, depending on the degree and nature of a patient's hand motor recovery at the time of stimulation.

It is conceivable that delivering both Anodal and Cathodal TDCS simultaneously via a bilateral M1–M1 electrode montage might, by summing both effects, produce greater benefits than either unilateral intervention. This logic has guided several studies using Bilateral TDCS which have reported transient changes in MEPs and improved hand motor performance in healthy controls and stroke patients (Fusco et al., 2013; Lefebvre et al., 2012; Lindenberg et al., 2010; Mahmoudi et al., 2011; Vines et al., 2008). However, for standard TDCS protocols (e.g.: 1 mA, 20 min), the assumption that a bilateral montage induces opposing physiological effects on M1 in a simple summative fashion (i.e.: excitability increase under the anode plus decrease under the cathode) has not yet been validated (although see Mordillo-Mateos et al., 2012). Moreover, while the effects of Anodal and Cathodal TDCS on contralateral hand excitability are well-established, effects on the ipsilateral hand are not routinely investigated. Any such ipsilateral effects might further complicate the impact and interpretation of bilateral stimulation. Here we aimed to subject the simple summative model of Bilateral TDCS to empirical test. First, we assessed the relative physiological efficacy of Bilateral versus Anodal and Cathodal TDCS in changing MEPs in the healthy brain, and then in changing paretic hand performance in chronic motor stroke.

A second aim of this study was to explore possible predictors of TDCS response. The many sources of heterogeneity in stroke populations, such as lesion size and site, symptom severity, residual anatomy, chronicity and pre-morbid status, entail variability in therapeutic outcome. In clinical practice, occupational, physical and drug therapies are routinely tailored to the individual patient to optimize therapeutic potential. Hence, there is incentive to identify clinical predictors or biomarkers that can explain outcome variability, and thus have predictive utility in tailoring TDCS appropriately based on an individual patient's profile (Lindenberg et al., 2012; Stinear, 2010; Stinear et al., 2007).

TMS and DTI probes of corticospinal tract integrity are useful markers of patients' functional recovery potential in stroke (Heald et al., 1993; Stinear, 2010; Stinear et al., 2007). Here we aimed to identify markers that could predict patients' behavioural response to TDCS. To do so, we used a simple motor task designed to be sensitive to patients' clinical level of hand motor impairment, and aimed to improve task performance with the paretic hand using TDCS. As potential predictors, we quantified patients' stroke characteristics and clinical levels of hand motor impairment, and obtained magnetic resonance spectroscopy (MRS) measures of γ-amino butyric acid (GABA) from M1 in the stroke-affected hemisphere. GABA is a candidate biomarker of plastic potential in response to TDCS, because both Anodal and Cathodal TDCS transiently decrease M1 GABA levels (Stagg et al., 2009a). A reduction in motor cortical GABA has been linked both to motor learning rate (Stagg et al., 2011a) and stroke recovery potential (Clarkson et al., 2010; Liepert et al., 2000). In chronic stroke patients, a GABA agonist has been shown to reinstate clinical symptoms (Lazar et al., 2010). To date, studies in human stroke patients have used drugs or TMS measures to assay the GABA-ergic system. To our knowledge, this is the first study to use GABA-MRS imaging in stroke. Our goal was to test the potential of MRS-measured ipsilesional M1 GABA levels to predict patients' behavioural response to TDCS.

## Methods

### Experiment 1: Comparative effect of Anodal, Cathodal and Bilateral TDCS on motor-evoked potentials in the healthy brain

#### Participants

Thirteen healthy volunteers (8 females; mean age = 24.6; SD = 4.8), twelve right-handed and one left-handed were recruited after ethical approval (Oxfordshire REC Committee A 06/Q1604/2). One subject did not undergo Bilateral TDCS. All were neurologically normal, had no contraindications to stimulation, and gave written informed consent.

#### Experimental design

The goal was to test the relative efficacy of Bilateral TDCS compared to Anodal and Cathodal TDCS in changing motor corticospinal excitability. Participants underwent three TDCS conditions, each separated by at least one week, in counterbalanced order. In each session, MEPs were recorded at rest from the right and left hand muscles before and after TDCS. Analyses then compared the relative amplitude and duration of TDCS-induced MEP changes from each hand in each TDCS condition.

Since the majority of the population are right-handed, and since left hemisphere strokes tend to result in greater disability (due to impairment of the dominant hand), we applied TDCS in a manner consistent with left hemisphere stroke. Namely, in each TDCS condition the ‘active’ electrode was placed over M1 as follows: 1) Anodal: over left M1; 2) Cathodal: over right M1; 3) Bilateral: anode over left M1 and cathode over right M1. Hence, the goal of all three conditions was to induce a relative increase in excitability of circuits controlling the right hand (the would-be ‘stroke-affected’ hand), and/or a relative decrease in excitability in circuits controlling the left hand. Experiment 1 aimed to test the relative efficacy of the three TDCS montages in inducing this pattern of physiological change.

#### Transcranial direct current stimulation (TDCS)

A direct current stimulator (Magstim, Whitland, Dyfed, U.K.) delivered stimulation (1 mA, 20 min) through a pair of 5 × 7 cm rubber electrodes encased in saline-soaked sponges and fixed to the head with rubber bands. The M1 electrode was centred over standard scalp coordinates for M1, 5 cm lateral from the vertex, with the long axis oriented anterior-posteriorly. For Anodal and Cathodal stimulation the reference electrode was placed over the contralateral supraorbital ridge.

#### Transcranial magnetic stimulation (TMS)

The following procedure was followed in each TDCS condition. First the ‘motor hotspot’ was localized for left and right M1, the optimal scalp position at which TMS evoked a just-noticeable twitch from the relaxed contralateral first dorsal inter-osseous (FDI) muscle. Next we determined at that location for each individual the minimum TMS intensity required to evoke ~ 1.5 mV MEPs from each contralateral FDI muscle in 10/10 trials. This TMS intensity yielded the baseline measure of motor corticospinal excitability and was kept constant throughout the experiment. There was one baseline and four post-TDCS blocks separated by 5 min intervals. In each block (~ 5 min), 40 single TMS pulses (20 per hand) were delivered in a pseudo-random order to left and right M1.

TMS was applied to each M1 through a pair of 70-mm figure-of-eight shaped TMS coils attached to a pair of monophasic TMS stimulators (Magstim Company, Whitland, Dyfed, U.K.). For two participants we could not fit both coils on the head, so one 50 mm coil was substituted. Both coils were held tangentially to the skull, fixed with metal clamps, and positional stability was monitored throughout.

#### Motor-evoked potentials (MEPs)

Electromyographic activity was recorded from each FDI muscle using neonatal electrocardiographic electrodes in a tendon-belly montage with a ground electrode on each elbow. Participants were told to relax their hand muscles during the experiment and compliance was monitored based on the background EMG. Responses were sampled, amplified and filtered using a CED 1902 amplifier, a CED micro1401 Mk.II A/D converter, and a PC running Spike2 (Cambridge Electronic Design). Signals were sampled at 5000 Hz with 50Hz notch filtering and band-pass filtered between 10 and 1000 Hz (Buch et al., 2011).

### Experiment 2: Comparative effect of Bilateral versus Anodal, Cathodal and Sham TDCS on motor performance with the paretic hand in chronic stroke

#### Patients

Thirteen chronic stroke patients (3 females, mean: 66 years, range 30–80 years) with hemiparesis subsequent to first-ever unilateral stroke were recruited after ethical approval (Oxfordshire REC Committee C 09/H0606/45) and written informed consent. Patient 13 did not undergo Bilateral TDCS. All were right-handed prior to the stroke. None of the lesions encroached directly on M1. Patients were screened to rule out contraindications to brain stimulation and personal or family history of psychiatric or neurological disease. Patient demographics, clinical status and stroke characteristics are in Inline Supplementary Table S1.

Inline Supplementary Table S1

**Table S1:** Individual patient characteristics. The table lists patients' percentage changes in reaction time (%ΔRT) in each TDCS condition (Real–Sham TDCS) and their scores on each predictor variable, as described in Experiment 3. The table is rank-ordered by ‘time since stroke’ and Upper Extremity Fugl–Meyer (UEFM) score, to facilitate comparison with Fig. 3D. Cortical lesions did not encroach on primary motor cortex. WTMF: Wolf Test of Motor Function. ^⁎^Patient 6's stroke hemisphere was mis-reported in Stagg et al. (2012). ^†^haem. = haemorrhagic stroke. N/A = not available.

Case	Anodal %ΔRT	Bilateral %ΔRT	Cathodal %ΔRT	Time since stroke (years)	UEFM	WTMF	Stroke side	Sex	Age	Stroke type	Lesion location	Lesion volume
9	0.72	5.28	− 8.60	5.8	66	75	right	F	30	^†^haem.	cortical	40398
5	− 36.43	− 33.34	− 19.07	5.3	40	54	left	M	66	infarct	subcortical	8141
7	− 23.11	− 36.47	− 22.85	5.2	57	63	left	M	62	infarct	cortical	102407
8	− 17.17	− 5.44	− 5.02	3.5	59	40	left	M	58	infarct	subcortical	1112
13	− 23.14	N/A	− 24.32	3.5	24	15	left	M	63	infarct	cortical	10220
1	− 6.05	− 0.21	− 9.66	3.3	49	35	right	M	62	infarct	subcortical	30
10	2.24	5.51	− 0.56	3.0	63	70	left	M	80	infarct	subcortical	671
12	− 8.03	0.01	16.63	2.9	62	64	right	M	66	infarct	subcortical	390
4	− 2.93	− 29.69	− 6.06	2.9	16	3	left	F	75	infarct	subcortical	2640
^⁎^6	− 20.35	2.12	− 10.76	2.8	35	41	right	M	71	infarct	subcortical	370
11	− 14.60	− 7.30	− 7.03	2.0	24	10	left	F	78	infarct	cortical	8091
3	− 0.83	− 5.53	− 2.01	1.8	40	42	left	M	78	infarct	cortical	N/A
2	− 8.81	10.50	8.11	1.5	27	16	right	M	74	infarct	cortical	237084

Inline Supplementary Table S1

#### Experimental design

The goal was to test the relative efficacy of Bilateral TDCS versus Anodal, Cathodal and Sham TDCS in improving paretic hand function. Patients underwent four TDCS conditions (min. 1 week intervals) in which they performed a simple reaction time (RT) task with the paretic hand before and after TDCS. Analyses focused on the percentage change in RT within-session (%ΔRT post-pre TDCS) compared to the Sham condition. Results for Anodal and Cathodal TDCS versus Sham have been reported previously, in a behavioural pilot experiment conducted prior to a separate fMRI study comparing these three conditions (Stagg et al., 2012). Here we tested the behavioural effect of Bilateral TDCS and report the comparative contrast with Anodal, Cathodal and Sham TDCS.

#### TDCS

Stimulation was applied identically to Experiment 1, in a single-blind fashion with patients blind to TDCS condition. Anodal TDCS was applied to ipsilesional M1, Cathodal to contralesional M1, and Bilateral to M1–M1 (anode-ipsilesional, cathode-contralesional). During sham stimulation the current was ramped up over 10 s, held constant at 1 mA for 15 s, and then ramped down over 10 s. With this procedure it has been shown that stroke patients and healthy volunteers cannot distinguish reliably between real and Sham TDCS (Gandiga et al., 2006).

#### Behavioural testing

Full details are in Stagg et al. (2012). Briefly, in each TDCS condition, patients performed blocks of the RT task with their paretic hand. The task was to make a joystick wrist flexion response as quickly as possible whenever a green circle appeared on a computer screen. Patients performed four baseline blocks, four blocks during TDCS and two blocks after TDCS. As there was no difference in performance during versus after TDCS, these six blocks were combined for analysis (all ‘post-TDCS’). RT task blocks were interleaved with blocks of a grip force task, but as TDCS had no effect on grip force we do not discuss it here.

### Experiment 3: Relationship between motor cortex neurochemistry and behavioural response to TDCS

#### Participants

A subset of eight patients from Experiment 2 (Inline Supplementary Table S1) and a group of age-matched healthy volunteers (3 females; mean age = 60, SD = 12.8) underwent magnetic resonance spectroscopy (MRS). Following ethical approval (Oxfordshire REC Committee A 06/Q1604/2) all were screened for contraindications to brain stimulation and imaging and gave written informed consent.

#### Experimental design

The goal was to test for a predictive relationship between ipsilesional M1 neurochemistry and behavioural response to TDCS. Participants underwent one MRS session in which levels of γ-amino butyric acid (GABA) and Glx (a composite measure of glutamate and glutamine) were measured first in ipsilesional M1 and then in a control region (occipital cortex). To determine whether neurochemical levels in M1 were abnormal post-stroke, patient data were compared to the group of age-matched healthy controls.

#### Magnetic resonance spectroscopy (MRS)

##### MRS acquisition

A 3 T Siemens/Varian MRI system was used. Sagittal and axial T1-weighted scout images (TR/TE = 3000/5 ms, TI = 1 ms, FOV = 512 × 256, matrix = 256 × 128) were acquired to place a 2 × 2 × 2 cm voxel of interest over the left precentral knob, a known landmark for hand motor representation (Yousry et al., 1997). In a separate acquisition session, a 2 × 3 × 2 cm control voxel was placed over the occipital cortex centred along the AC–PC line on an axial slice. To assess the creatine and N-acetyl aspartate (NAA) linewidths, a standard PRESS sequence (TR/TE = 3000/68 ms) was used to acquire an unedited spectrum with 32 averages. Edited GABA spectra with 256 averages were acquired from the voxel of interest using the MEGA-PRESS sequence (TR/TE = 3000/68 ms) with 20 ms double-banded Gaussian inversion pulses for simultaneous spectral editing and water suppression (Mescher et al., 1998). The water suppression band was set to a frequency of 4.7 ppm, and an editing band alternated between 1.9 ppm (edit on) and 7.5 ppm (edit off) in even and odd acquisitions.

##### MRS analysis

MRS data analysis was performed using jMRUI v2.2 (http://www.mrui.uab.es/mrui/). Each spectrum was analysed by two independent observers with high inter-rater reliability (α = 0.89). Prior to fitting in jMRUI, data were corrected for any non-zero DC offset, smoothed using a 2 Hz Lorentzian filter, and had zero and first order phase corrections applied. The residual water signal was removed using a Hankel Lanczos singular value decomposition (HLSVD) filter.

Creatine line-widths were obtained from the non-edited PRESS acquisition using AMARES, a non-linear least square fitting algorithm (Vanhamme et al., 2001), and was used to constrain the linewidths of the GABA and glutamate/glutamine (Glx) resonances from the edited spectra. Both GABA and Glx resonances were fitted with 2 Gaussian peaks. A single Gaussian curve was fitted to the NAA resonance and was constrained to the linewidth of NAA in the non-edited spectrum. Spectra with an NAA linewidth of greater than 10 Hz were excluded from further analysis.

FMRIB's Automated Segmentation Tool (FAST), part of the FMRIB software library (www.fmrib.ox.ac.uk/fsl), was used on the T1-weighted structural scan to calculate the relative quantities of grey matter and white matter within the voxel of interest as reported previously (Stagg et al., 2009a). All neurotransmitter concentrations are given as a ratio of NAA and have been corrected for number of equivalent protons, and grey to white matter tissue fraction.

### Statistical analysis

Statistical analyses on data from all experiments were conducted using SPSS v.21 (IBM Corp.). Since Levene's test for equality of variances revealed that variance in the MRS data differed between patients and controls, a non-parametric Mann–Whitney *U* test was used to compare MRS data between patients and controls. MEP and RT data were analysed using RM ANOVA with Huyhn–Feldt correction and planned contrasts using one-sample or paired samples t-tests, as appropriate. Pearson's R correlations were uncorrected for multiple comparisons. Simple and stepwise multiple linear regression was used to test for predictors and to determine the specificity of observed correlations. The significance level was p < .05.

## Results

### Experiment 1: Comparative effects of Anodal, Cathodal and Bilateral TDCS on MEPs in healthy volunteers

Mean MEP amplitudes were calculated separately for each participant, hand and block (Pre, Post 1–4) in each TDCS session (Anodal, Cathodal, Bilateral). Data were normalized for each individual by calculating the mean percentage change in MEP amplitude (%ΔMEP) in each Post-TDCS block relative to the baseline.

**Fig. S1:**
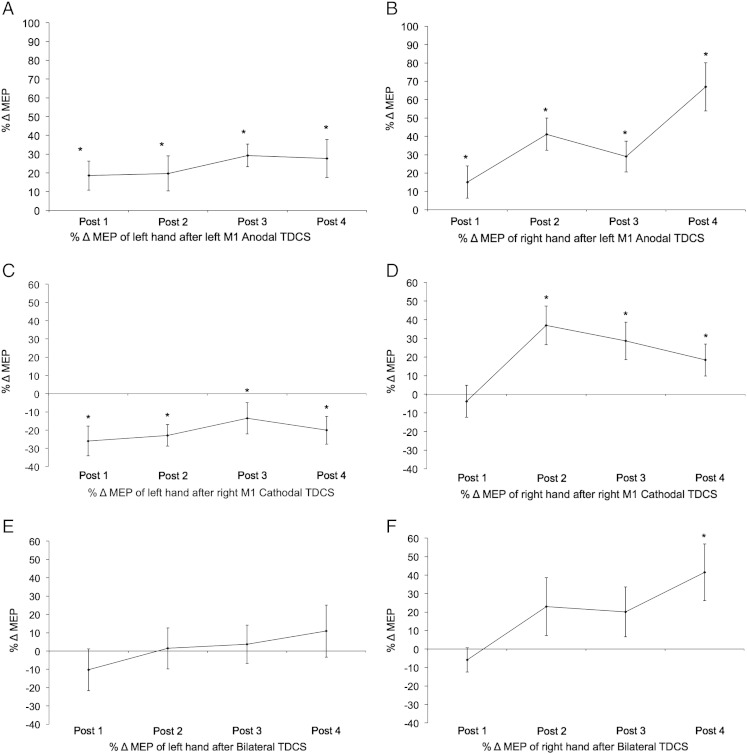
MEP timecourses. Figure shows group mean percentage changes in MEP amplitudes (%ΔMEP) from each hand in each block after TDCS. Error bars = ± 1 SEM. Asterisks indicate a significant difference from zero (1-sample *t*-test, p < .05 uncorrected for multiple comparisons).

To test the hypothesis that TDCS would change MEP amplitude in a manner that varied by both stimulation polarity and hand, RM ANOVA was conducted on %ΔMEP data with factors of TDCS (Anodal, Cathodal, Bilateral), Hand (Right, Left) and Time (Post 1, 2, 3, 4). All main effects were significant (TDCS [F(2,22) = 11.055, p < .001], Hand [F(1,11) = 14.092, p = .003] and Time [F(3,33) = 10.099, p < .001]) as well as the Hand × Time interaction [F(3,33) = 3.352, p = .031] (all else n.s., see Inline Supplementary Fig. S1). Since we had no hypothesis about Time, the data were pooled over Time (creating a mean %ΔMEP score post-TDCS) and a follow-up RM ANOVA re-confirmed the main effects of both TDCS (p < .001) and Hand (p < .001).

Inline Supplementary Figure S1

**Fig. 1:**
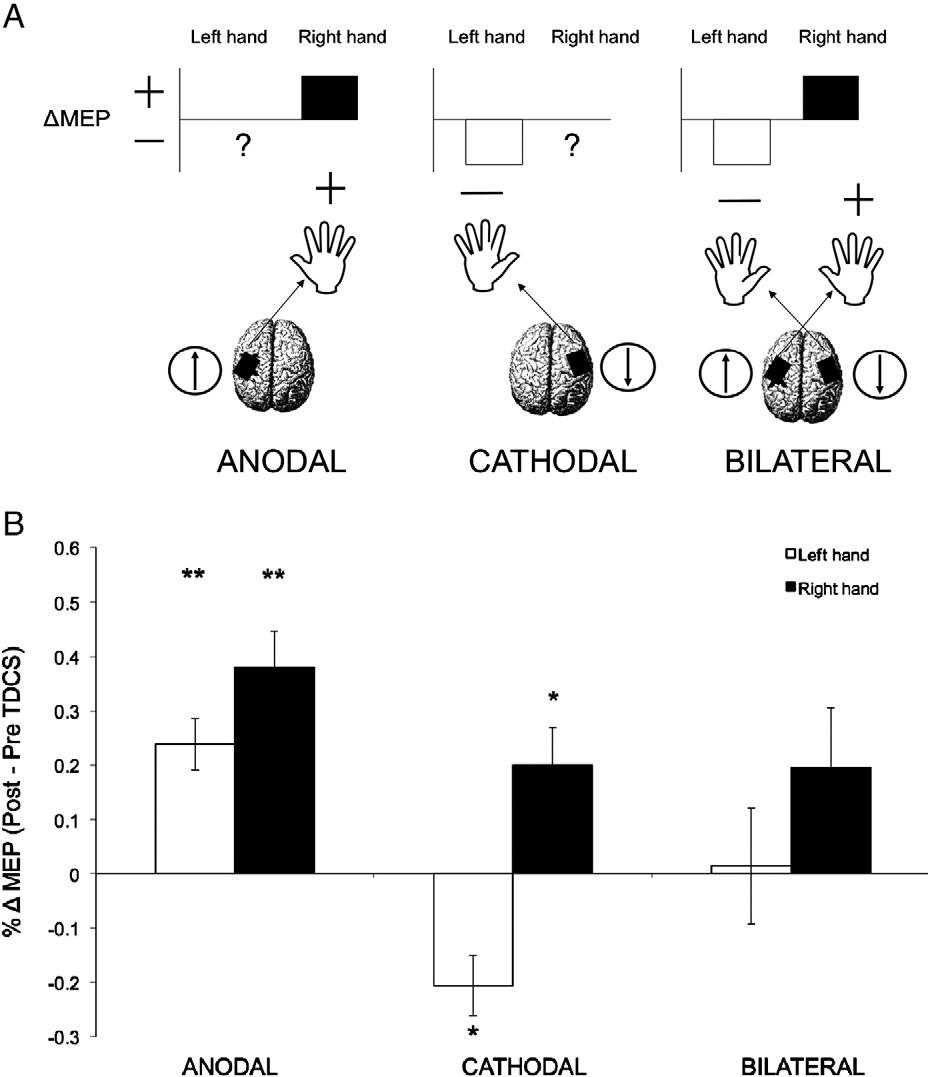
Comparative effects of Anodal, Cathodal and Bilateral TDCS on motor evoked potentials (MEPs) in healthy volunteers. A) Hypothesis: The effect of Bilateral TDCS would be the sum of the Anodal and Cathodal conditions. The figure illustrates the hypothesis that the constituent effects of the anode and cathode on the contralateral hand would sum together in the Bilateral M1–M1 electrode montage, thus causing an increase in MEPs from the right hand and a decrease in MEPs from the left hand. B) Comparative effects of Anodal, Cathodal and Bilateral TDCS. The graph shows the observed percentage change in the amplitude of MEPs (%ΔMEP) from each hand in each TDCS condition. Anodal TDCS induced a correlated MEP increase from both hands. Cathodal TDCS decreased MEPs from the contralateral left hand and increased MEPs from the ipsilateral right hand. These two effects were not correlated. Contrary to predictions, the effects of Bilateral TDCS were not summative: there were no significant MEP changes. Columns represent mean ± 1SEM. Significant mean changes are represented by asterisks (*p < .05, **p < .001).

We further investigated these two main effects as follows. First, we decomposed the data by the factor of TDCS and carried out a series of one-sample t-tests (against zero) to test whether, within each TDCS condition, there was a significant change in the excitability of each hand (Fig. 1). Second, we decomposed the data by the factor of Hand, and conducted one-way RM ANOVAs, one per hand, to compare the relative efficacy of each TDCS condition in inducing a relative excitability increase in the right hand and/or a relative excitability decrease in the left hand.

In the Anodal condition, as expected, mean excitability of the contralateral (right) hand (A_contra_) increased (t(12) = 5.812, p < .001; M = 38%, SD = 23.5). Unexpectedly, there was a similar increase in the ipsilateral (left) hand (A_ipsi_) (t(12) = 5.002, p < .001; M = 24%, SD = 13.5) and the excitability increases across the two hands were correlated (Pearson's R(13) = .624, p = .023).

In the Cathodal condition, we observed the expected decrease in excitability of the contralateral (left) hand (C_contra_) (t(12) = − 3.715, p = .003; M = − 21%, SD = − 19.9) accompanied by an increase in excitability of the ipsilateral (right) hand (C_ipsi_) (t(12) = 2.917, p = .013; M = 20%, SD = 24.7). These two effects were not correlated (Pearson's R(13) = .698, p = .119).

In the Bilateral condition, we hypothesized that the M1–M1 electrode montage would increase excitability of the right hand (contralateral to the anode) and decrease excitability in the left hand (contralateral to the cathode). However, neither effect was significant (Right Hand: t(11) = 1.818, p = .096; M = 19.6%, SD = 37.4; Left Hand: t(11) = .136, p = .895; M = 1.5%, SD = 37.1) or inter-correlated (p = .2).

To test the relative efficacy of the three TDCS conditions, two one-way RM ANOVAs were conducted on the mean %ΔMEP data, one per hand.

For the right hand, the pattern did not vary by electrode montage (F(2,22) = 2.106, p = .146), reflecting a tendency for MEPs to increase in all three TDCS conditions. However, planned contrasts revealed a significant difference between the Anodal and Bilateral conditions (t(1,11) = 5.272, p = .042; difference M = 19%, SD = 28.8), indicating that Anodal TDCS induced a larger excitability increase than Bilateral TDCS. By contrast, the unexpected excitability increase in the ipsilateral (right) hand after Cathodal TDCS did not differ in amplitude from the expected excitability increase in the contralateral (right) hand after Anodal TDCS (t(1,11) = 3.365, p = .094).

**Fig. S2:**
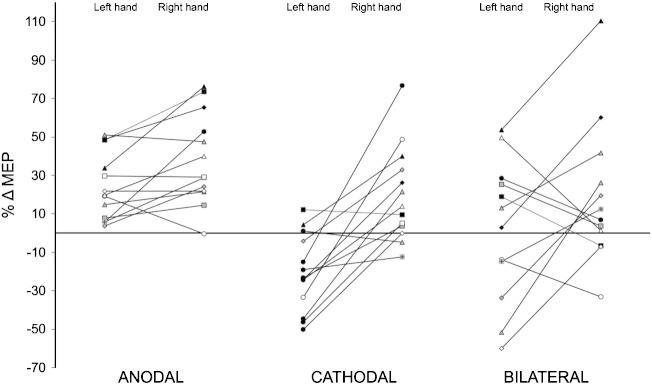
Inter-individual variation in MEP effects. Figure shows individual differences in percentage changes in MEP amplitudes (%ΔMEP) from each hand in each TDCS condition.

For the left hand, the pattern of MEP change varied by TDCS condition (F(2,22) = 11.846, p < .001). Planned contrasts confirmed a significant difference between the left hand excitability decrease after Cathodal and increase after Anodal TDCS (t(1,11) = 50.487, p < .001; difference M = 44.3%, SD = 21.6). The Bilateral condition also differed from the Cathodal condition (t(1,11) = 5.323, p = .042; difference M = − 19.8%, SD = 29.8), reflecting the fact that left hand excitability was unchanged after Bilateral stimulation. Individual variability in %ΔMEP data is plotted in Inline Supplementary Fig. S2.

Inline Supplementary Figure S2

#### Predicting MEP response to Bilateral TDCS

Although Bilateral TDCS did not induce significant within-session changes in excitability, we aimed to determine whether the pattern of %ΔMEP data in the Bilateral condition could be predicted from responses in the Anodal and Cathodal conditions. Thus we aimed to test whether, in the Bilateral montage, the anode did in fact drive the response of the contralateral right hand, and the cathode the response of the contralateral left hand.

**Fig. 2:**
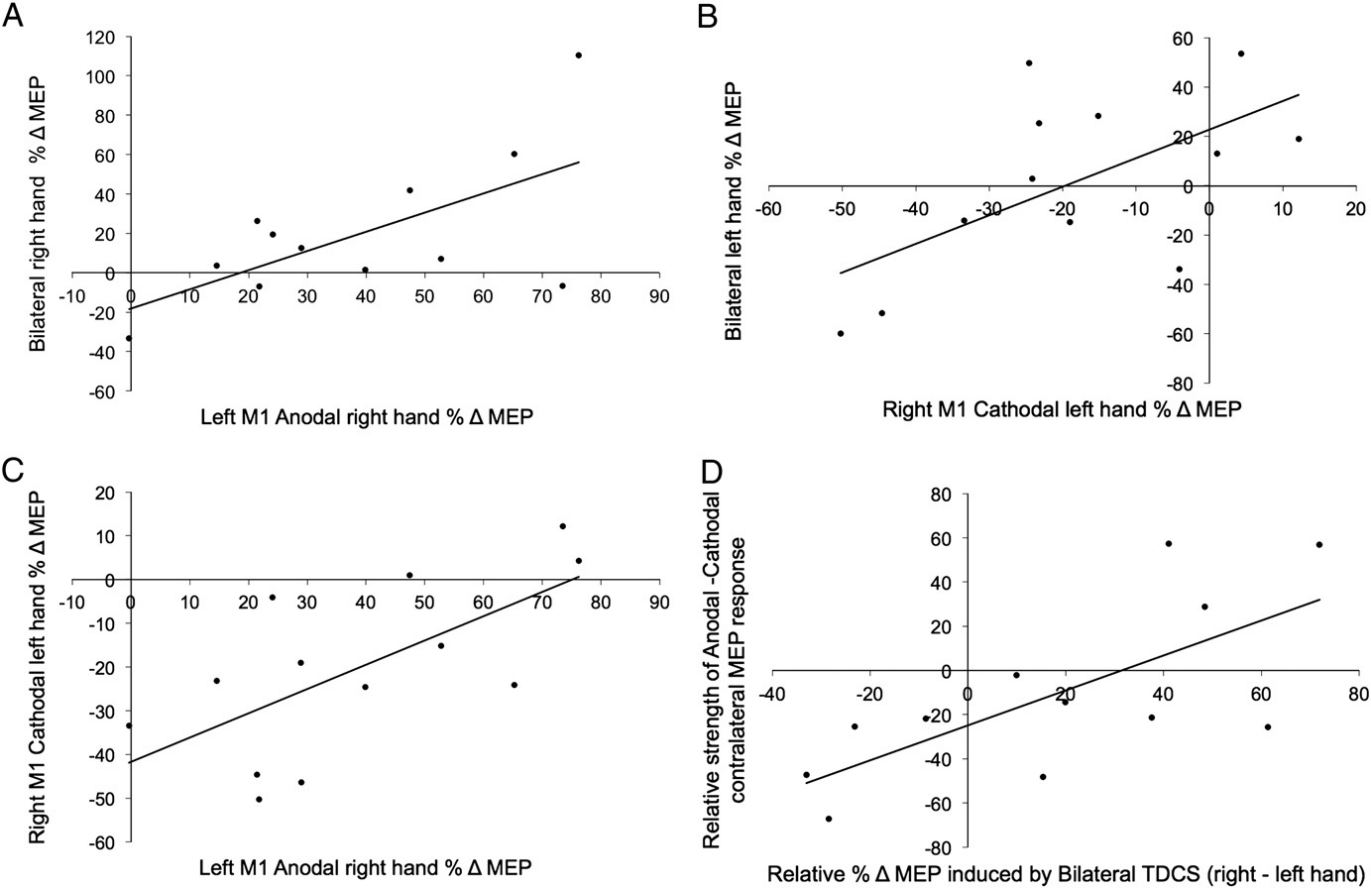
Predicting the response to Bilateral TDCS from the constituent effects of Anodal and Cathodal TDCS. Plots show percentage MEP changes after TDCS (%ΔMEP). Regression analyses confirmed that individuals' response to Bilateral TDCS could be predicted from their response to the conventional unilateral Anodal and Cathodal TDCS conditions. A) The increase in contralateral (right) hand MEP amplitude in the Anodal condition predicted the right hand response to Bilateral TDCS. B) The decrease in contralateral (left) hand MEP amplitude in the Cathodal condition predicted the left hand response to Bilateral TDCS. C) The amplitude of contralateral hand MEP changes was inversely correlated across individuals. D) Depending on whether an individual showed a relatively stronger Anodal or Cathodal contralateral hand MEP response, this determined which of the two electrodes would dominate the overall response to Bilateral TDCS.

We used simple linear regression to investigate a possible predictive relationship in the %ΔMEP data between: 1) Anodal contralateral (right) hand and Bilateral right (B_right_) hand responses; 2) Cathodal contralateral (left) hand and Bilateral left (B_left_) hand responses. Both predictors were significant. The Anodal contralateral (A_contra_) hand excitability increase predicted the B_right_ hand response (Fig. 2A) (adjusted R square = .346: F(1,10) = 6.82, p = 0.026; *β* = .637) and this remained significant after removal of two influential outliers in the B_right_ hand condition (adjusted R square = .519: F(1,8) = 10.71, p = 0.011; *β* = .757). The Cathodal contralateral (C_contra_) hand excitability decrease predicted the B_left_ hand response (Fig. 2B) (adjusted R square = .296: F(1,10) = 5.623, p = 0.039; *β* = .600). Changes in Anodal or Cathodal ipsilateral hand excitability did not predict the response of either hand to Bilateral TDCS (all p > .05).

The %ΔMEP data for A_contra_ and C_contra_ hands were inversely correlated (Pearson's R(13) = .656, p = .015) (Fig. 2C). That is, for a given individual, the stronger the A_contra_ hand excitability increase, the weaker the C_contra_ hand excitability decrease. Given that the rationale underlying the Bilateral montage was to combine the opposing A_contra_ and C_contra_ hand effects, this suggested that the overall pattern of response to Bilateral TDCS might be determined by the relative strength of each constituent effect.

To test this, first we quantified each individual's relative contralateral hand responsivity to Anodal versus Cathodal TDCS (%ΔMEP A_contra_ − C_contra_). Next we derived a new variable that would capture the degree to which Bilateral TDCS drives the excitability of the two hands in opposite directions. This variable, expressing the right and left hand change in excitability with Bilateral TDCS, was calculated as: %ΔMEP B_right_ − B_left_ hand. The correlation between relative responsivity to Anodal versus Cathodal TDCS (%ΔMEP A_contra_ − C_contra_) and relative response across the two hands to Bilateral TDCS (%ΔMEP B_right_ − B_left_ hand) was significant (Pearson's R(12) = .703, p = .011) (Fig. 2D). That is, whether the right (contralateral to the anode) or left (contralateral to the cathode) M1 effect dominated in the Bilateral montage depended on individuals' relative responsivity to each of the constituent effects. This single regressor (%ΔMEP A_contra_ − C_contra_) was a significant predictor of response to Bilateral TDCS for both the B_right_ (adjusted R square = .347: F(1,10) = 6.849, p = 0.026; *β* = .638) and B_left_ hands (adjusted R square = .385: F(1,10) = 7.887, p = 0.019; *β* = .664).

### Experiment 2: Relative effect of Bilateral versus Anodal, Cathodal and Sham TDCS on simple RT with the paretic hand in chronic stroke

Results for the comparison of Anodal and Cathodal TDCS versus Sham have been reported previously (Stagg et al., 2012). Here we report the comparison of Bilateral versus Anodal, Cathodal and Sham TDCS. RT means and SDs were calculated for each block and each patient after outlier removal (RTs > 2 s or ± 2SD). Mean RTs were then normalized for statistical analysis, first quantified within-session as percentage change from baseline (%ΔRT), and then across conditions as %ΔRT with respect to the Sham condition. RM ANOVA indicated no difference in baseline RT across the four TDCS conditions, so the grand mean was computed (M = 576 ms, SD = 232) and used as a regressor in Experiment 3.

**Fig. 3:**
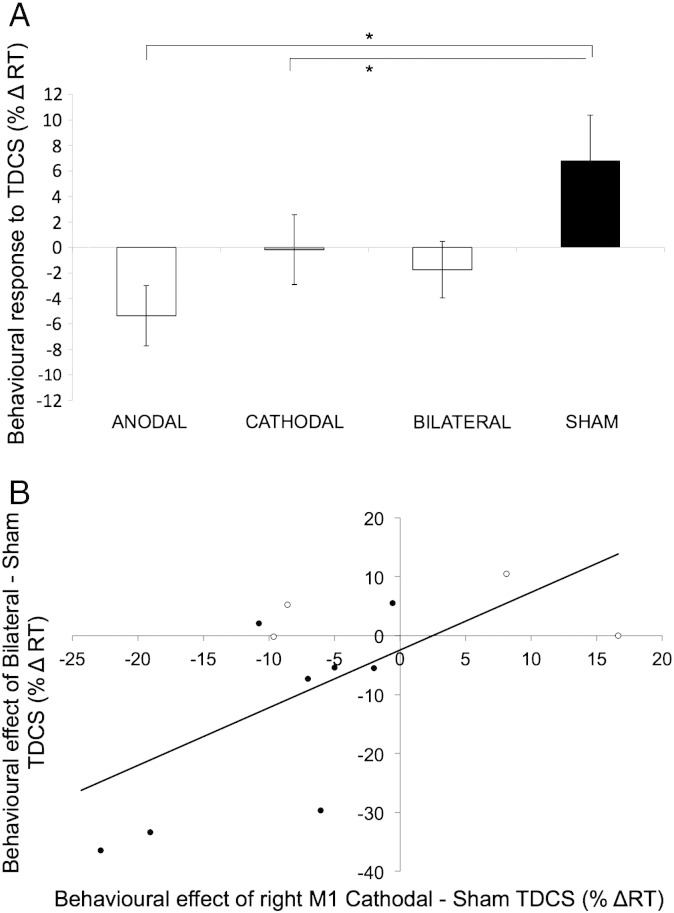
Comparative effect of Bilateral versus Anodal, Cathodal and Sham TDCS on simple reaction time with the paretic hand in chronic stroke. A) Whereas both Anodal and Cathodal TDCS improved reaction time relative to Sham Bilateral TDCS had no effect. Data are partially re-drawn from Stagg et al. Cortical activation changes underlying stimulation-induced behavioural gains in chronic stroke. Brain. 2012. 135, 276–284, by permission of Oxford University Press. B) Patients' behavioural response in the Cathodal condition was a significant predictor of their response to Bilateral TDCS. Data are percentage change in reaction time after TDCS (%Δ RT). Unfilled symbols represent patients with right hemisphere strokes.

RM ANOVA on the within-session % ΔRT data, with factors of TDCS (Anodal, Cathodal, Bilateral, Sham) and stroke hemisphere (right, left), revealed a main effect of TDCS (F(3,30) = 3.506, p = .027) and an interaction (F(3,30) = 3.039, p = .044). Planned contrasts revealed that the TDCS × Hemisphere interaction was significant only for the contrast of Bilateral − Sham (F(1,10) = 6.402, p = .03). In the Sham condition, patients exhibited a fatigue effect (RT increase over time, Fig. 3A) that did not differ between hemispheres (p < .05). This fatigue effect was reduced after Bilateral TDCS in patients with left hemisphere strokes (t(6) = − 2.546, p = .044 difference M = − 16%, SD = 16.6), but not in those with right hemisphere strokes (t(4) = 1.772, p = .151) (Fig. 3B; compare open and filled symbols). However, analysis of the within-session %ΔRT data revealed that there was no change in task performance before versus after Bilateral TDCS in either group (p < .1).

As reported previously (Stagg et al., 2012, Brain), both Anodal (p = .002) and Cathodal TDCS (p = .048) speeded RT significantly compared to Sham (Fig. 3A). As in Experiment 1, we next tested whether individuals' response to Anodal or Cathodal TDCS would predict their response to Bilateral TDCS. To quantify the effect of TDCS, we focussed analyses on the contrast of the within-session %ΔRT in each real TDCS condition minus the Sham condition. The within-session %ΔRT was unrelated across the four conditions (all p > .05). These analyses revealed positive correlations between the effects of Anodal and Cathodal TDCS (Pearson's R(12) = .600, p = .03), Cathodal and Bilateral TDCS (Pearson's R(12) = .648, p = .023) and a trend between Anodal and Bilateral TDCS (Pearson's R(12) = .571, p = .053), suggesting that individual differences in the degree to which TDCS reduced fatigue were somewhat consistent across electrode configurations. Multiple linear regression confirmed that the combination of Anodal and Cathodal regressors could significantly predict the Bilateral data (enter method: adjusted R square = .371: F(2,11) = 4.250, p = .05, *β_Anodal_* = .308, *β_Cathodal_* = .478), but the Cathodal condition alone was the better predictor of behavioural response to Bilateral TDCS (stepwise method: adjusted R square = .362: F(1,10) = 7.229, p = 0.023; *β* = .648) (Fig. 3B).

### Experiment 3: Relationship between patients' M1 GABA levels and behavioural response to TDCS

The goal was to test the hypothesis that ipsilesional M1 GABA levels would predict the behavioural response to TDCS described in Experiment 2. First, we tested whether neurochemistry (levels of GABA:NAA, Glx:NAA and NAA:Cr) in ipsilesional M1 differed between patients and age-matched controls (Mann–Whitney *U* test: all p > .13, n.s.). Next, to test the hypothesis that ipsilesional M1 GABA:NAA levels would predict behavioural response to TDCS, we computed correlations between these neurochemical measures and: 1) baseline task performance; 2) the change in performance with TDCS (within-session %ΔRT); and 3) the effect of TDCS relative to Sham (between-session %ΔRT).

Correlations between M1 neurochemical levels and baseline task performance (grand mean basal RT across all four conditions) were not significant (all p < .2). For the within-session %ΔRT data, only the correlation with the Sham condition was significant (Pearson's R(8) = .724, p = .042; all else p > .05). That is, the higher a patient's ipsilesional M1 GABA:NAA levels, the greater their fatigue on the task.

**Fig. 4:**
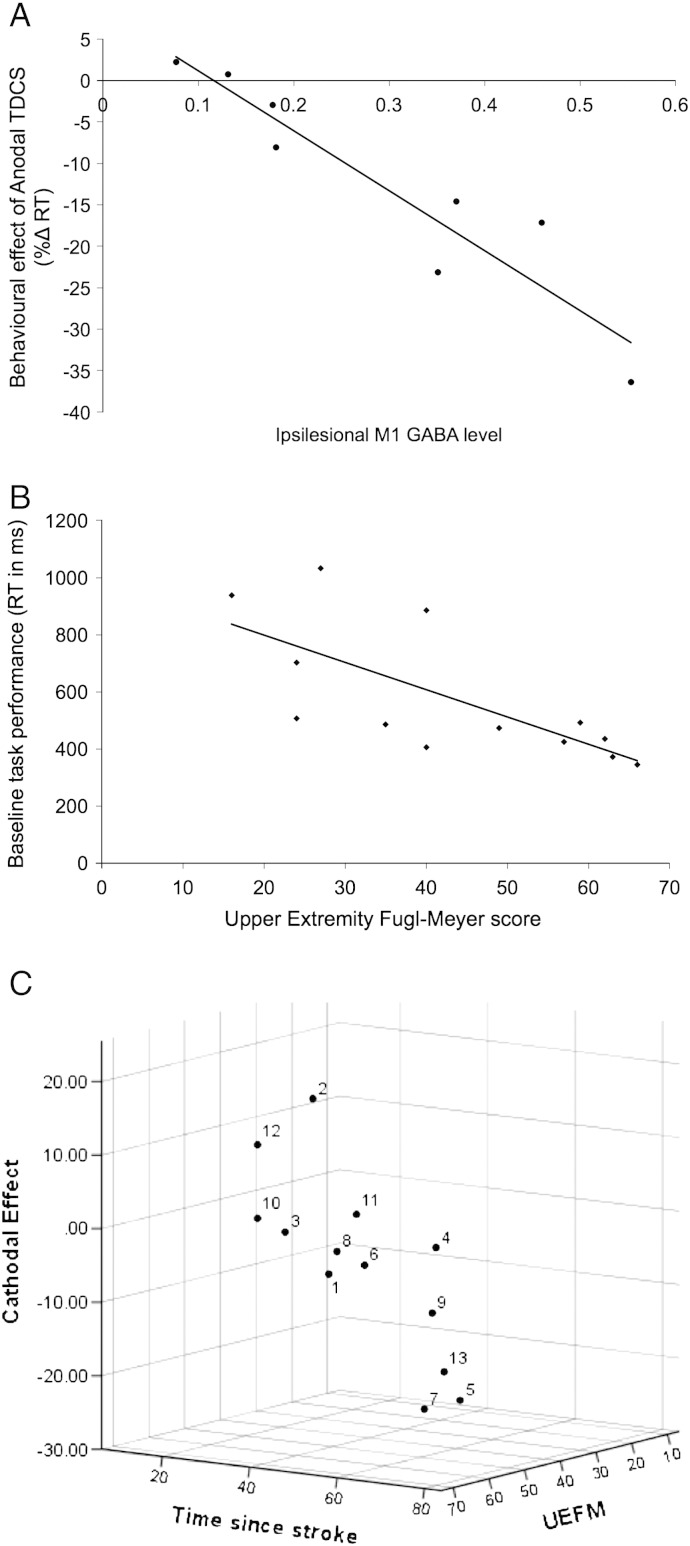
Predicting patients' behavioural response to Anodal and Cathodal TDCS. Regression analyses revealed significant predictors of patients' behavioural response to Anodal and Cathodal TDCS. A) M1 GABA levels predicted the Anodal effect. The higher the level ratio of GABA:NAA in patients' ipsilesional motor cortex (M1), the larger their reaction time gain in response to Anodal TDCS. B) The better a patient's paretic hand function (UEFM score), the faster their baseline performance of the simple RT task. C) Patients' response to Cathodal TDCS was best predicted by a 2-factor regression model that combined ‘time since stroke’ and UEFM score. That is, the longer the time since a patient's stroke and the better their recovery, the greater their RT gain in response to Cathodal TDCS. By contrast, those patients with a shorter time since stroke who had made a poorer recovery showed a smaller RT gain or an RT deficit in response to Cathodal TDCS. Numbers list individual cases (Inline Supplementary Table S1). UEFM = Upper Extremity Fugl–Meyer score (max = 66). All regressions are significant (all p < .05).

For the between-session %ΔRT data, ipsilesional M1 GABA levels predicted response to Anodal TDCS (Pearson's R(8) = − .936, p = .001). That is, the higher a patient's ipsilesional M1 GABA levels, the greater their RT gain with Anodal TDCS compared to Sham (Fig. 4A). The correlation with M1 Glx:NAA was also significant (Pearson's R(8) = − .775, p = .024), likely reflecting the inter-correlation between M1 GABA:NAA and Glx:NAA levels (Pearson's R(8) = .871, p = .005). To determine neurochemical specificity, we computed partial correlations to assess the relationship of each neurochemical with behaviour while controlling for the other. The partial correlation between the Anodal RT gain and M1 GABA levels remained significant (Pearson's R(5) = − .841, p = .018), while the correlation with M1 Glx:NAA did not (Pearson's R(5) = .240, p = .604). The M1 GABA:Anodal RT gain correlation remained significant after controlling for basal RT (p = .003). M1 NAA:Cr levels did not correlate with behaviour or with GABA or Glx levels. A multiple linear regression model confirmed that M1 GABA:NAA was a unique predictor of the behavioural response to Anodal TDCS (adjusted R square = .855, F(1,6) = 42.292, p = 0.001; *β* = − .936, p = .001).

Ipsilesional M1 GABA:NAA levels did not predict the behavioural response to Cathodal (Pearson's R(8) = − .532, p = .175) or Bilateral TDCS (Pearson's R(8) = − .543, p = .164). Neither was there any correlation with M1 Glx:NAA or M1 GABA:Glx in either condition (all p > .05).

Finally, there was no relationship between V1 GABA:NAA or Glx:NAA levels and baseline RT or %ΔRT data in any TDCS condition (all p > .05), confirming the anatomical specificity of the results for M1.

#### Clinical predictors of behavioural response to TDCS in motor stroke

Baseline performance on the simple RT task (Experiment 2) correlated with Upper Extremity Fugl–Meyer (UEFM), indicating that patients with poorer paretic hand function performed the simple RT task more slowly (Pearson's R(13) = − .708, p = .007) (Fig. 4B). Hence, the simple RT task was sufficiently sensitive to capture clinically meaningful inter-individual variation in paretic hand function.

To test whether clinical status, demographic or stroke characteristics could predict the behavioural response to TDCS, we carried out regression analyses on the variables listed in Inline Supplementary Table S1.

There was no relationship between any predictor variable and behavioural response to Anodal or Bilateral TDCS (p > .05).

‘Time since stroke’ correlated with the Cathodal TDCS effect (%ΔRT Real − Sham TDCS) (Pearson's R(13) = − .567, p = .043). That is, the longer the time since a patient's stroke, the larger their RT gain from Cathodal stimulation. Step-wise multiple linear regression revealed that a two-factor model combining ‘time since stroke’ with UEFM better predicted the data (adjusted R square = .528: F(2,10) = 7.719, p = 0.009; *β_Time_* = − .866, p = .003; *β_UEFM_* = .612, p = .023) (Fig. 4C).^3^ The model indicates that those patients with greater chronicity and better paretic hand function showed greater RT gains in response to Cathodal TDCS. By contrast, patients who had had their stroke more recently and who had poorer paretic hand function showed either a smaller RT gain or an RT decrement. Hence, Cathodal TDCS to the contralesional hemisphere tended to benefit patients who had made a good recovery over time, but was less effective or even deleterious for patients with more recent strokes who had recovered less well.

## Discussion

This study aimed to contrast the physiological and behavioural effects of different TDCS electrode montages, and to test whether predictors of TDCS response could be identified. By contrast with Anodal and Cathodal stimulation, Bilateral TDCS neither changed MEPs in the healthy brain nor improved simple reaction time with the paretic hand in chronic stroke. Hence, Bilateral M1–M1 TDCS was less effective than unilateral Anodal or Cathodal TDCS. We also aimed to identify potential predictors of patients' behavioural response to TDCS. Ipsilesional M1 GABA levels predicted reaction time gains from Anodal TDCS. Patients' response to Cathodal TDCS over contralesional M1 was best predicted by a two-factor model combining time since stroke with Fugl–Meyer score. Overall, these findings demonstrate the superiority of Anodal or Cathodal TDCS over Bilateral TDCS and identify candidate predictors of patient outcome variability in response to TDCS intervention for hemiparesis in chronic stroke.

Contrary to predictions, the Bilateral M1–M1 electrode montage did not combine in a simple summative fashion the constituent effects of the anode and cathode on excitability of the contralateral hand (Fig. 1). In fact, there was no significant change in MEPs measured from either hand. Importantly, however, correlation analyses confirmed that the %ΔMEP data in the Bilateral condition could be predicted from responses in the Anodal and Cathodal conditions. Thus, the Bilateral data could be explained both by the response to each constituent electrode (Figs. 2A,B) and their combination (Figs. 2C,D). In combination, these findings indicate that the Bilateral montage did indeed stimulate both M1s in the expected direction, but the effects were weak and variable.

Bilateral TDCS was also ineffective in changing reaction time with the paretic hand in stroke patients (Fig. 3A). Whereas Anodal–ipsilesional and Cathodal–contralesional M1 TDCS improved RT significantly relative to Sham, Bilateral TDCS did not. More accurately, when Bilateral TDCS was applied to patients with left hemisphere strokes, it reduced the fatigue observed in the Sham condition, with no such effect in right-hemisphere stroke patients (Fig. 3B). Notably, therefore, the reliable RT facilitation observed in the Anodal condition was abolished in the Bilateral montage. Correlation analyses showed that the between-session RT change (real-Sham TDCS) tended to correlate across Bilateral, Anodal and Cathodal conditions. This suggests that the current did indeed stimulate the underlying motor cortices in the Bilateral TDCS condition in a manner somewhat similar to the other real TDCS conditions. Hence, consistent with the MEP results, the bilateral montage produced weaker and more variable effects, and was sub-optimal compared with either conventional unilateral montage.

The relative inefficacy of 1 mA Bilateral versus unilateral TDCS in changing M1 excitability has been reported before, albeit with a shorter stimulation duration (Nitsche and Paulus, 2000). This lesser efficacy may be related to current shunting across the scalp or to complex inter-hemispheric interactions during M1–M1 stimulation. Previous reports of positive findings for Bilateral TDCS likely reflect key differences in protocol. In their MEP study Mordillo-Mateos et al. (2012) used double the current (2 mA). Two patient studies combined Bilateral TDCS with motor training over one (Lefebvre et al., 2012) or more sessions (Lindenberg et al., 2010) and with longer duration stimulation than that used here (1 mA versus 1.5 mA, both for 30 min). Neither study assessed relative efficacy versus Anodal or Cathodal TDCS. Vines et al. (2008) reported that Bilateral TDCS with large electrodes and the opposite polarity to that used here (anode over right M1, cathode over left M1) improved motor sequence learning in right-handers performing with their left hand. The relative ease of inducing physiological and behavioural effects of Anodal, Cathodal and Bilateral may depend on hemispheric dominance. Consistent with this, in our study, Bilateral TDCS was even less effective in right-hemisphere than left-hemisphere stroke patients. Future work on Bilateral TDCS should investigate the importance of hemispheric dominance, current strength and duration and address relative efficacy versus Anodal and Cathodal TDCS, in order to determine the optimal protocols to take forward to clinical trials.

Although Experiment 1 was mainly motivated by a wish to compare Bilateral TDCS to conventional configurations, it also produced novel findings regarding Anodal and Cathodal TDCS. Whilst the effect of TDCS on contralateral MEPs is well characterized, few studies have assessed ipsilateral MEPs. We found that both stimulation polarities changed MEPs bilaterally. Anodal TDCS increased the excitability of both hands in a correlated fashion. Cathodal TDCS decreased excitability contralaterally and increased excitability ipsilaterally, and these two effects were not correlated. In addition, we found an inverse correlation between individuals' response to Anodal versus Cathodal TDCS: individuals who showed a large Anodal increase in contralateral (right) hand excitability showed a small Cathodal decrease in contralateral (left) hand excitability, and vice versa. Future work could test whether the effects observed here differ with hemispheric dominance or handedness, and whether the induced changes in ipsilateral MEPs have functional consequences.

A major goal of this study was to identify potential clinical or neurochemical predictors of behavioural response to TDCS. Such markers could help to identify candidate stroke patients likely to benefit from TDCS. We distinguish two apparent forms of behavioural effect observed here. In the Sham condition, patients exhibited a slowing of RT over the course of the experiment, likely reflecting fatigue (Fig. 3A). There was an overall main effect of TDCS, whereby stimulation reduced this fatigue. This global effect was polarity-independent, suggesting a rather non-specific effect of stimulation, such as increased arousal, led to this reduction in fatigue. Note that such an effect, although rather unspecific, may still be of interest for rehabilitation (e.g.: by enabling longer duration physiotherapy sessions). By contrast, specifically in the Anodal condition, there was a significant reduction in RTs after versus before TDCS, with patients performing the task significantly faster after TDCS not only with respect to the Sham session, but also with respect to the Anodal baseline session. Two observations suggest that this reflects a genuine functional facilitation specific to Anodal TDCS. First, the magnitude of individual patients' performance improvement could be quantitatively predicted by GABA levels in ipsilesional M1. Second, in our prior fMRI study, a similar behavioural facilitation was observed, and the magnitude of behavioural change correlated with the magnitude of fMRI signal change in the stimulated ipsilesional M1. In combination, these two findings suggest a genuine gain in task performance caused by Anodal TDCS, arising through a specific interaction with individual patients' residual neurochemistry in M1 circuits controlling the stroke-affected hand.

In our patient cohort, variation in ipsilesional M1 GABA:NAA levels predicted variation in reaction time gains from Anodal TDCS (Fig. 4A). This effect was both neurochemically and anatomically specific, since there was no correlation with M1 Glx:NAA and no correlation with GABA:NAA in occipital cortex. Patients with higher ipsilesional M1 GABA levels exhibited larger RT gains from Anodal TDCS. How might this be interpreted? We have shown previously, in healthy controls, that Anodal TDCS induces a local reduction in M1 GABA (but not Glx) levels (Stagg et al., 2009a). Those with high M1 GABA levels may therefore have a higher potential dynamic range for GABA modification by Anodal TDCS. Although our previous studies in healthy volunteers did not find evidence of correlation between basal M1 GABA and its modification by Anodal TDCS (Stagg et al., 2011a), this does not rule out the possibility of such a relationship existing in stroke patients. Future studies could test this by measuring GABA pre and post TDCS.

In our previous work on healthy volunteers, higher M1 GABA levels were associated with slower manual reaction times (Stagg et al., 2011b). In the present study, that relationship was not significant, although inspection of the data suggests that this may simply reflect small sample size, since patients with high GABA:Glx ratios tended to be slower at the task. Importantly, however, the relationship between GABA levels and Anodal RT gain remained significant after controlling for basal RT. Baseline task performance correlated with Fugl–Meyer score, confirming that the task used here is a clinically meaningful probe of patient's residual or recovered paretic hand function. Hence, just as DTI and TMS measures of corticospinal tract integrity are useful indicators of post-stroke functional potential (Stinear and Ward, 2013), so ipsilesional M1 GABA levels may prove a useful biomarker of patients' plastic potential in response to Anodal TDCS.

Although patients' M1 GABA levels did not differ from age-matched controls, this may simply reflect low power. M1 TMS studies suggest that intracortical inhibition is reduced acutely post-stroke (Liepert et al., 2000), and may (Swayne et al., 2008) or may not (Blicher et al., 2009) re-normalize at the chronic stage, likely depending on injury severity and time since stroke. It would be interesting to investigate co-variation in MRS and TMS measures of M1 intra-cortical inhibition over time post-stroke, since these methods appear to assay distinct aspects of GABA-ergic function (Stagg et al., 2011b). GABA-MRS may offer a means to assess recovery potential in patients from whom MEPs cannot be evoked.

Variability in patients' response to Cathodal TDCS was best predicted by a two-factor model combining ‘time since stroke’ with Fugl–Meyer score (Fig. 4B). The regression model showed that Cathodal TDCS tended to benefit those patients who had made a good recovery over many years since their stroke, but was less effective, or even deleterious, for patients with more recent strokes who had recovered less well. This coheres with existing fMRI and TMS evidence on patterns of motor activity with functional recovery, in which less impaired patients might be expected to benefit from Cathodal TDCS, which should help to ‘re-normalize’ activity to the ipsilesional hemisphere. By contrast, in poorly-recovered patients, who may require bilateral motor activity to compensate for their injury, Cathodal TDCS may be less effective or even disruptive (Bradnam et al., 2012; Johansen-Berg et al., 2002; Ward et al., 2003b). Consistent with this, inspection of individual patient data shows that patient 4, who had the longest time since stroke (5.8 years) and the maximum possible UEFM score (66), showed an 8.6% RT improvement in response to Cathodal TDCS. By contrast, patient 5, who had the shortest time since stroke (1.5 years) and a low UEFM score (27), showed an 8.1% RT deficit in response to Cathodal TDCS. However, the comparison of patients 6 and 9, who had near-identical ‘time since stroke’ and UEFM scores is also instructive. Patient 6 showed a negligible response to Cathodal TDCS, while patient 9 showed a 16.6% RT decrement. This suggests that the functional significance of activity in contralesional M1 differed for these two patients, a factor not captured by either predictor variable. Other measures, such as TMS probes, could provide useful additional information to determine the functional status of contralesional motor activity and hence potential suitability for Cathodal TDCS (Fridman et al., 2004; Johansen-Berg et al., 2002; O'Shea et al., 2007). A related point is that TDCS effects are not confined to the directly stimulated M1, but are known to also change functional signal in premotor and supplementary motor regions (Stagg et al., 2009b, 2012). The interaction between TDCS-induced changes in the directly stimulated M1, together with induced changes in functional activity in regions within (and outside) the wider motor network may be critical determinants of the behavioural outcome for paretic hand function.

The present study aimed to identify potential clinical and neurochemical markers that could be usefully incorporated into future clinical trials of TDCS to help explain patient outcome variability. Neurochemical and clinical predictors with moderate to high regression effect sizes were identified, suggesting that these variables have potential predictive utility. However, due caution should be applied in extrapolating from the relatively small patient sample size tested here (N = 8/13). In addition, these measures predicted TDCS-induced change in a simple behavioural task. To induce long-term clinically meaningful behavioural change, it is likely that TDCS will need to be paired with a more complex behavioural intervention (e.g.: physiotherapy), with the aim of using (Anodal) TDCS to enhance acquisition or consolidation of the training effects (Reis et al., 2009). Whether ipsilesional M1 GABA levels or clinical measures will have similar predictive utility when TDCS is combined with these more complex behavioural interventions remains to be investigated.

Overall, this study demonstrates the superiority of Anodal or Cathodal TDCS over Bilateral TDCS in changing cortical excitability and motor behaviour and identifies candidate predictors of patients' plastic response to TDCS. The identified predictors, if validated in larger samples, could help to explain inter-individual variability in behavioural response to TDCS, and thus prove useful in future TDCS stroke trials.

## Footnotes

3 Since the two clinical scores (UEFM and WTMF) were inter-correlated (Pearson's R(13) = .910, p < .001), but the WTMF also correlated with ‘time since stroke’ (Pearson's R(13) = .592, p < .033), only UEFM was tested in the regression model.
