# Supplementary material for: Predicting behavioural response to TDCS in chronic motor stroke
Source: Neuroimage. 2014 Jan 15;85(Pt 3):924–33. doi: 10.1016/j.neuroimage.2013.05.096 (PMC3899017; doi:10.1016/j.neuroimage.2013.05.096)
Supplement: Inline Supplementary Table S1 [file mmc1.doc]

**Table 1. Patient characteristics**

| Case | Anodal %ΔRT | Bilateral %ΔRT | Cathodal %ΔRT | Time  since  stroke  (years) | UEFM | WTMF | Stroke  side | Sex | Age | Stroke  type | Lesion  location | Lesion volume |
| --- | --- | --- | --- | --- | --- | --- | --- | --- | --- | --- | --- | --- |
|  |  |  |  |  |  |  |  |  |  |  |  |  |
| 9 | 0.72 | 5.28 | -8.60 | 5.8 | 66 | 75 | right | F | 30 | †haem. | cortical | 40398 |
| 5 | -36.43 | -33.34 | -19.07 | 5.3 | 40 | 54 | left | M | 66 | infarct | subcortical | 8141 |
| 7 | -23.11 | -36.47 | -22.85 | 5.2 | 57 | 63 | left | M | 62 | infarct | cortical | 102407 |
| 8 | -17.17 | -5.44 | -5.02 | 3.5 | 59 | 40 | left | M | 58 | infarct | subcortical | 1112 |
| 13 | -23.14 | N/A | -24.32 | 3.5 | 24 | 15 | left | M | 63 | infarct | cortical | 10220 |
| 1 | -6.05 | -0.21 | -9.66 | 3.3 | 49 | 35 | right | M | 62 | infarct | subcortical | 30 |
| 10 | 2.24 | 5.51 | -0.56 | 3.0 | 63 | 70 | left | M | 80 | infarct | subcortical | 671 |
| 12 | -8.03 | 0.01 | 16.63 | 2.9 | 62 | 64 | right | M | 66 | infarct | subcortical | 390 |
| 4 | -2.93 | -29.69 | -6.06 | 2.9 | 16 | 3 | left | F | 75 | infarct | subcortical | 2640 |
| *6 | -20.35 | 2.12 | -10.76 | 2.8 | 35 | 41 | right | M | 71 | infarct | subcortical | 370 |
| 11 | -14.60 | -7.30 | -7.03 | 2.0 | 24 | 10 | left | F | 78 | infarct | cortical | 8091 |
| 3 | -0.83 | -5.53 | -2.01 | 1.8 | 40 | 42 | left | M | 78 | infarct | cortical | N/A |
| 2 | -8.81 | 10.50 | 8.11 | 1.5 | 27 | 16 | right | M | 74 | infarct | cortical | 237084 |
|  |  |  |  |  |  |  |  |  |  |  |  |  |

**Supplementary Table 1. Individual patient characteristics.** The table lists patients’ percentage changes in reaction time (%ΔRT) in each TDCS condition (Real – Sham TDCS) and their scores on each predictor variable, as described in Experiment 3. The table is rank-ordered by ‘time since stroke’ and Upper Extremity Fugl-Meyer (UEFM) score, to facilitate comparison with Fig. 4C. Cortical lesions did not encroach on primary motor cortex. WTMF: Wolf Test of Motor Function. *Patient 6’s stroke hemisphere was mis-reported in Stagg et al. 2012. † haem. = haemorrhagic stroke. N/A = not available
